# Use of Linguistic Complexity in Writing Among Chinese EFL Learners in High-Stakes Tests: Insights From a Corpus of TOEFL iBT

**DOI:** 10.3389/fpsyg.2021.765983

**Published:** 2021-10-28

**Authors:** Leyi Qian, Yan Cheng, Yali Zhao

**Affiliations:** School of Foreign Studies, Hefei University of Technology, Hefei, China

**Keywords:** high-stakes test, linguistic complexity, TOEFL iBT, topic effects, writing

## Abstract

In studies on second language writing, linguistic complexity exhibited by learners has long been regarded as being indicative of writing proficiency. However, there are relatively scant studies focusing on the diversity and structural elaboration of complexity in L2 production data that are extracted from high-stakes tests [such as Test of English as a Foreign Language (TOEFL) and International English Language Testing System (IELTS)]. Using a large-scale learner corpus collected from a TOEFL (internet-based test (iBT), this study aims to explore the extent to which the three dimensions of linguistic complexity, syntactic, lexical, and morphological complexity, are associated with human scoring in high-stakes tests. In addition, we also tend to tap into within-genre topic effects on the production of complexity measures by learners. To this end, a total of 1,002 writing samples were collected from a TOEFL11 corpus, and six automated-coding instruments were used to investigate the variations of complexity among Chinese English as a Foreign Language (EFL) learners. The results from the correlation analysis, multiple linear regression, and independent sample *t-*tests indicated that there was not a linear correlation between the majority of linguistic complexity and human-rated score levels and that proficiency among Chinese EFL learners did not signal a discriminative power in their language production. In the meantime, strong within-proficiency topic effects were found on the majority of measures in the syntactic, lexical, and morphological dimensions.

## Introduction

As one of the two productive skills (the other is speaking), writing constitutes an essential part of education. Among the indicators of writing quality, the presence of linguistic complexity has received extensive attention among L2 researchers and practitioners, since indices of complexity have been considered of vital importance in evaluating language production and can be used “to gauge proficiency, to describe performance, and to benchmark development” (Ortega, 2012, p.128). Meanwhile, studies have shown that it is more difficult to write linguistically complex sentences than linguistically simple ones (e.g., Wolfe-Quintero et al., 1998; Bastiaanse et al., 2009). In addition, a good command of linguistic features can facilitate L2 learners to enhance writing skills, such as planning, drafting, and revising (Sasaki, 2000; Cumming, 2001). Therefore, among the errors committed by language learners, the presence or absence of linguistic complexity deserves closer observation, as understanding the extent to which learners make use of linguistic elements in written contexts will offer a holistic and accurate picture with respect to the linguistic repertoire that is desired for a proficient writer in academic settings.

The global spread of English in academic contexts has led to its pervasive power over EFL/ESL education around the world. One manifestation lies in the increasing number of students who are registering for and taking international standardized tests like TOEFL and IELTS as proof of their language qualifications and as their first step to embark on adventures in education overseas. However, despite best efforts, Chinese test-takers still display poor performance in TOEFL Writing Section as evidenced by the *Test and Score Data Summary for TOEFL iBT® Test* (2010–2019). Although there has been improvement in TOEFL holistic score from 77 points in 2010 to 81 points in 2019 (on a total score of 120), the average score of writing is 20 points (on a score scale of 30), a score which falls within the range of *High*-*Intermediate* level (17–23) and remains unchanged over the past 10 years (with the only exception in 2010, reaching 21 points)^1^. This poses no optimistic prospect to their endeavors in pursuing further studies overseas as most programs, especially graduate programs in the United States, require a considerably higher level of English proficiency. Accordingly, there is a pressing need to address writing problems displayed in writing skills among Chinese EFL learners in TOEFL-like high-stakes tests.

With this in mind, our study is, therefore, aimed to extend previous studies using a large-scale learner corpus collected from TOEFL iBT to explore the variations in the production of complexity measures by learners at different human-rated writing score levels along the syntactic, lexical, and morphological dimensions, as well as the effects of topic on the selection of linguistic features. The findings will provide insights into what linguistic devices are truly predicative of and would correlate with quality writing in academic settings: a real linking of “complexity” theory with practice.

## Linguistic Complexity: An Overview

As a complex and integrated skill, writing deploys a combination of linguistic qualities that are desirable in a text and considered essential for L2 learners attempting to combine language with their ideas and thoughts. Among the linguistic features in academic writing, complexity, coupled with accuracy and fluency, which target factors such as correctness and speed, has been examined in a large body of studies to assess L2 proficiency and development. As a multi-dimensional and multi-componential construct, complexity has been divided into absolute and relative complexity (Bulte and Housen, 2012). Relative complexity is related to psychological or cognitive complexity, i.e., cost and difficulty of processing or learning; In contrast, absolute complexity is representative of “objective properties of linguistic units.” According to their taxonomy, absolute complexity consists of three components: propositional complexity, discourse-interactional complexity, and linguistic complexity, where linguistic complexity is further distinguished *via* system complexity and structure complexity. System complexity deals with a lexical layer in the language system and its subsystems, engaging variables such as collocational and lexemic items; while structure complexity is composed of formal and functional complexity targeting morphological (inflectional, derivational) and syntactic (sentence, clause, and phrase) properties, respectively. In this article, we adopt the definition and taxonomy proposed by Bulte and Housen (2012) in which complexity can be captured by the numbers, length, range, and diversity displayed through grammatical structures such as syntactic, lexical, and morphological items.

Among the three dimensions of linguistic complexity, syntactic complexity features a prominent status in the research on L2 writing, since it has been regarded as one of the essential factors that contribute to second language proficiency and has been used to indicate more proficient writing. As a quality of language output (Ryshina-Pankova, 2015), syntactic complexity has been examined from various perspectives in the literature, and a wide range of indices have been the target of quantifications. Broadly speaking, the operationalization of complex measures in the syntactic layer can be categorized at three different levels (sentence, clause, and phrase), each of which is geared toward a designated aspect of syntactic complexity. In addition, four major parameters can be used as quantitative indications to account for the variations of complexity: length, ratio, index, and frequency (Norris and Ortega, 2009), among which length, ratio, and frequency are the most common indicators.

Another construct that locates the properties of complexity reflecting the developmental progression of the learner is on the lexical spectrum. Although quite a number of studies have been conducted to investigate lexis-related variables, perception of the dimensions of lexical complexity varies across L2 researchers and practitioners. In this article, we adopt the taxonomy proposed by Bulté and Housen (2014) in which lexical complexity as an umbrella term comprises lexical diversity and lexical sophistication. The former is primarily either ratio-based or index-based like type/token ratio (TTR), CTTR (Corrected TTR, Carroll, 1964), Guiraud index (types/square root of tokens) (Guiraud, 1960); the latter is related to the lexical knowledge that manifests itself in a wide variety of words used in a successfully written L2 text. In other words, lexical diversity equates complexity with the density or proportion of lexical items that are incorporated into syntactic structures. In contrast, lexical sophistication is suggestive of non-repetitious or different lexical items in writing.

As a layer of language structure, morphology bridges the gap between meaning and its function, in which roots and affixes of words constitute the building block of morphological competence (Pirrelli et al., 2015). In addition, morphological profiling has proven to be vital to the L2 development and learning process (Bardovi-Harlig, 1999; Prévost and White, 2000).

Until now, a number of indicators of morphological complexity, such as verb placement, frequency of tensed forms, verbal inflection, morphological derivation, have been proposed for its measurement (Malvern et al., 2004; Pirrelli et al., 2015). Other predictors of complexity in morphology include the Types per Family (T/F) index (Horst and Collins, 2006), the measure of Inflectional Diversity (ID; Malvern et al., 2004), the mean size of verbal paradigms (Xanthos and Gillis, 2010) and morphological complexity index (MCI) (Brezina and Pallotti, 2015; Pallotti, 2015).

However, although there are quite a number of studies along the line of linguistic complexity, to the best of our knowledge, no research until now has been conducted to examine the writing of students in high-stakes tests in terms of all the structural dimensions of complexity. It should be highlighted that due to distinctions between tests and real-life academic writing, such as time allocation, text length, resource accessibility, audience (Riazi, 2016) as well as the degree of pressures, devotion, and seriousness, there can be apparently varying evidence regarding the writing performance of students in different writing situations. Such lack of study has motivated this study, as writing in academic settings is typically designed to produce written texts to meet the expectations of academic institutions (Paltridge, 1994; Berkenkotter and Huckin, 1995; Connor, 1996), where students can complete the assigned tasks in universities or colleges to demonstrate the acquired knowledge in related courses (Hale et al., 1996; Waters, 1996).

## Relationship Between Linguistic Complexity and Writing Quality

Complexity measures have been adequately and objectively quantified among L2 writing researchers to predict and account for the variation in writing development of learners on the ground that complex linguistic forms in L2 production have been considered to be indicative of writing quality and could predict the holistic writing scores of learners in the process of language learning. To date, a number of studies have been carried out on the contribution of varied mastery of linguistic complexity to writing quality (e.g., Parkes and Zimmaro, 2016; Yoon, 2017), and on how linguistic complexity can influence L2 teaching and L2 development (Ellis and Yuan, 2004; Abedi and Gandara, 2006; Mazgutova and Kormos, 2015), as well as the role genre plays in the assessment of writing performance among EFL learners (Qin and Uccelli, 2016; Jeong, 2017; Olson et al., 2018; Amini and Iravani, 2021).

Among the predictive indices of linguistic complexity, the ability to use more linguistically complex syntactic structures in a foreign language can be suggestive of foreign language development (Ortega, 2012) and distinguish between L2 proficiency levels (Wolfe-Quintero et al., 1998). Quite a number of studies have analyzed the relationships between measures of syntactic complexity and L2 writing quality. For instance, metrics, such as words per clause (Beers and Nagy, 2009), T-unit based measures (Ortega, 2003; Kang and Lee, 2019), clause-level complexity (Grant and Ginther, 2000; Taguchi et al., 2013), and syntactically complex phrases (Yang et al., 2015; Biber et al., 2016; Staples and Reppen, 2016), have been found to correlate positively with high quality of writing performance.

As such, studies on either longitudinal or cross-sectional designs have also yielded mixed findings concerning the relationship between measures of syntactic complexity and writing scores (e.g., Ortega, 2003; Crossley and McNamara, 2014), indicating that patterns of syntactic development are not closely and consistently aligned with ratings. Additionally, different writing performances and linguistic features have been observed from task-related variables like effects of between-discourse-mode (Way et al., 2000; Lu, 2011) and topics within the same discourse mode (Yang et al., 2015; Yoon, 2017).

Engagement in a wide range of lexical measures can also be regarded as a yardstick to assess the ability of L2 learners to use English and detect possible lexical deficiencies. For instance, positive correlations have often been reported between more diverse lexical items and higher holistic scores in written discourses (e.g., Cumming et al., 2005; Zareva et al., 2005; Yu, 2010; Kim, 2014; Karakoc and Kose, 2017). In addition, findings related to lexical sophistication have indicated that using lexical measures is critical in shaping both first and second language development (Duran et al., 2004; Yoon, 2018; Vogelin et al., 2019), and can discriminate proficiency levels in SLA (Jarvis, 2002; Crossley and McNamara, 2010; Treffers-Daller et al., 2018). However, although cross-sectional studies have been carried out to demonstrate that genre plays a discriminative role in extracting lexis-related variability (e.g., Olinghouse and Wilson, 2013; Amini and Iravani, 2021), little research has been dedicated in the literature to investigate the role assigned topics have played in contributing to choices of words of learners in their writings. Meanwhile, mixed findings have also been yielded in terms of the contribution of lexical measures to the improvement in overall writing proficiency. For instance, Bulté and Housen (2014) found that higher values of lexical constructs failed to reach better writing quality among EFL students in an EAP program (English for Academic Purpose) spanning one semester. Likewise, no significant differences were observed in the study of Pietila (2015) on the relationship between proficiency levels and linguistic production of lexical knowledge by students.

Finally, because of the simple morphology of the English language (De Clercq and Housen, 2016), complexity in morphology has been rarely examined in writing research on L2 learners and on how morphological complexity can affect L2 language development and proficiency. Nevertheless, research in SLA has shown that morphological complexity can be used to discriminate between language proficiency levels and serve as a useful sub-construct characterizing linguistic complexity in the context of SLA (Verspoor et al., 2012; Bulté, 2013). Meanwhile, a significant developmental trend was also observed in first language acquisition (Malvern et al., 2004; Xanthos and Gillis, 2010), and positive correlations between morphological productivity and oral proficiency have also been observed (De Clercq and Housen, 2016). Notably, the only attempt to explore topic effects on morphological complexity revealed that the values of MCI can differ significantly across two argumentative topics (Yoon, 2017).

## The Study

Given the positive evidence that complexity has provided in measuring writing quality in most studies, as well as the conflicting findings that are revealed in some other studies, it is of vital importance to revisit and re-assess the role of complexity in contributing to the judgment of written texts in various academic settings. To date, there is only one study in the literature (Guo et al., 2013) that has investigated the predictive features of linguistic complexity in TOEFL iBT tests. However, their corpus size was rather limited, and the effects of proficiency and topic were not clarified. Moreover, its essays consisted of both integrated and independent writing samples, the findings of which would be less convincing, since, in integrated writing tasks, any failure to detect relevant information in the listening and reading materials will influence the performance of test takers and, therefore, cannot reliably represent their writing proficiency. What is more, as essential components of linguistic complexity, morphological measures were not touched upon in their study.

Motivated by the increasing amount of attention to the complexity and the scarcity of research on one of the two major world-renowned English proficiency tests (the other being the IELTS test) in this respect, we aim to explore the correlations between the constructs of complexity and writing scores in international standardized English proficiency assessments. As mentioned before, the production of such high-level academic tests would elicit different writing behaviors and demonstrate different language abilities among learners. In the meantime, high-stakes tests are expected to differ fundamentally from low-stakes, the ones that are administered in instructional settings, such as EAP programs, classroom practices, and writing coursework, and pose few challenges and are less difficult to students. Language learners in TOEFL-like test-driven measurement of writing proficiency are assumed to be sufficiently prepared and have given full play to the materials and writing techniques. In addition, high-stakes tests like TOEFL iBT can foster motivation and engagement of students in taking writing tasks instead of casual and careless, even unwilling, responses to fulfilling the measurement procedure imposed by researchers and practitioners. Based on the evaluative criteria for writing tasks in *TOEFL 2000 framework: A working paper* (Jamieson et al., 1999), two comprehensive perspectives have been highlighted. One is on a macro-level perspective, addressing issues related to the organization of discourse and ideas by ESL students. The other is on a micro-level perspective, addressing issues related to syntax, lexis, and morphology. Briefly, from a micro-level perspective, this study aims to explore the variables that deal with aspects of language use in TOEFL independent writing tasks. Specifically, this study seeks to address the following research questions:

1. What features of syntactic, lexical, and morphological complexity have high predictive power and value that are directly interpretable between proficiency levels? That is, how complexity governs the use of linguistic devices in TOEFL independent writing tasks among Chinese test takers. Furthermore, is human-assigned scoring significantly and positively correlated with linguistically complex measures?
2. How do topic-related variables affect the writing performance of test takers regarding the use of complexity at different levels and subjective evaluations by human raters?

We assume that there will be clear differences between writing quality and values of complexity-related measurement in relation to the syntactic, lexical, and morphological indices. Specifically, we expect that there would be a linear correlation between the three dimensions of linguistic complexity, namely, syntactic, lexical, and morphological, and human expert scorings of Chinese EFL writings. The growth in the manipulation of linguistic features would result in high scores in essays. Our further assumption is that the written production of complexity measures by learners can be affected by topic effect since topic familiarity would play an important role in explaining variances in the human global judgment of essay quality. In other words, different topics require different reasoning and cognitive demand imposed on writers, thus leading to the variability in the use of linguistically complex structures.

## Methods

### Corpus Data

Our selected corpus for this study included sample essays written by Chinese EFL learners collected from the Educational Testing Service (ETS) research report (ETS RR-13-24): TEOFL11: A Corpus of Non-Native Written English (Blanchard et al., 2013) (TOEFL11 corpus). The TOEFL11 corpus includes 12,100 essays written by international TOEFL iBT (Internet-Based Test) test-takers in 11 L1 non-English native languages (Arabic, Chinese, French, German, Hindi, Italian, Japanese, Korean, Spanish, Telugu, and Turkish), with a single essay for each examinee. Essays for each language were evenly sampled in the TOEFL11 dataset, totaling 1, 100 written samples collected from the TOEFL independent writing tasks of eight argumentative prompts, along with human scoring levels for each writing task response. According to this report (2014), each essay was first rated by highly trained human raters on a 5-point-scale and later collapsed into a 3-point-scale: *low* (scoring between 1 and 2), *medium* (scoring between 2.5 and 3.5), and *high* (scoring between 4 and 5). This study excludes essays rated as *low*, as they contain “a noticeably inappropriate choice of words or word forms,” “an accumulation of errors in sentence structure and/or usage”, and “serious and frequent errors in sentence structure or usage.”^2^ In addition, there were only 98 essays across eight prompts at the *low*-score level, and some *low-*scored essays are even <50 words in length. In all, a total of 727 essay samples from the group *medium* and 275 from the group *high* across eight prompts from Chinese test takers were selected. The distributions and descriptive statistics of the selected essays are shown in Tables 1, 2 shows the summary of all eight prompts.

**Table 1:** Distribution and descriptive statistics of the selected essays.

	***N***	**Essay length (Group** ***medium*****)**				***N***	**Essay length (Group** ***high*****)**			
**Prompts**		**Mean**	***SD***	**Minimum**	**Maximum**		**Mean**	***SD***	**Minimum**	**Maximum**
Prompt1	94	307.20	59.28	184	509	39	368.26	62.96	278	531
Prompt2	90	329.69	59.41	182	564	37	386.95	76.23	237	578
Prompt3	74	304.97	49.82	195	441	44	393.50	73.74	270	556
Prompt4	85	304.38	46.41	206	478	35	353.00	63.27	283	595
Prompt5	92	334.34	49.60	230	467	33	378.91	89.89	258	799
Prompt6	86	342.81	56.72	197	536	44	386.48	59.39	286	535
Prompt7	104	316.39	57.33	170	483	26	366.12	54.13	279	487
Prompt8	102	314.67	58.37	128	472	17	370.47	67.19	281	536
Total	727	319.44	56.43	128	564	275	377.00	69.73	237	799

**Table 2:** Summary of prompts.

**Do you agree or disagree with the following statement? Use specific reasons and examples to support your answer**.	
P1	It is better to have a broad knowledge of many academic subjects than to specialize in one specific subject.
P2	Young people enjoy life more than older people do.
P3	Young people nowadays do not give enough time to helping their communities.
P4	Most advertisements make products seem much better than they really are.
P5	In 20 years, there will be fewer cars in use than there are today.
P6	The best way to travel is in a group led by a tour guide.
P7	It is more important for students to understand ideas and concepts than it is for them to learn facts.
P8	Successful people try new things and take risks rather than only doing what they already know how to do well.

### Selection of Complexity Measures

Among the variety of syntactic indices, it has been pointed out that lengths of production units can be misleading and cannot serve as reliable and consistent metrics in analyzing complexification in varying layers of syntactic organization for the reason that length-based indexes collapse multiple syntactic features into a single variable (“omnibus” measure) [refer to Biber et al. (2020) for details]. In other words, although two sentences may share almost the same value regarding length per T-unit, they can be syntactically different in terms of the number of dependent clauses per T- unit and of prepositional phrases that modify nouns. As a result, a good candidate for the predicative power should take into consideration a single underlying feature that involves quantitative analysis, as omnibus measures fail to capture the structural and syntactic differences in the analysis of their contribution writing of learners. For this, we adopted the stance of Biber et al. (2020) on linguistic interpretability by excluding the measure of mean length of T-unit. We also excluded the index of mean length of sentence to avoid redundancy and confusion, since the clause should be taken as the base unit (Yang et al., 2015).

As a major manifestation of syntactic construct, clause-level complexity is generally assessed in terms of three indices: dependent clauses per clause (DC/C), clauses per sentence (C/S), and dependent clauses per T-unit (DC/T). The obvious overlap of DC/C with DC/T has led to the inclusion of only DC/T and C/S as the selected measures. In addition, there are overlaps between C/S and clause per T-unit (C/T) and T-unit per sentence (T/S), because C/S consists of both subordinate clauses and coordinate clauses, C/T is a measure for subordination, and T/S is a measure for coordination. To avoid repetitive measurement, we narrow down our attention to four clause-level indices that target clausal level complexity: DC/T, C/S, C/T, and T/S. As conceptualized in *TOEFL 2000 framework: A working paper* (Jamieson et al., 1999), evaluative criteria regarding NP complexity and participle phrase can be suggestive of variances in essay performance in independent writing tasks; therefore, the third set of measures relating to phrase-related variables included indices of coordinate phrase per T-unit (CP/T), complex nominal per T-unit (CN/T), and verb phrase per T-unit (VP/T).

Lexical complexity does not only refer to rich knowledge of vocabulary, but also to an appropriately organized knowledge of vocabulary. However, the majority of existing lexical measures in the literature have been primarily focused on quantifying observable lexical properties that can be traced in a text without exploring the relationships between such measures that are attributable to writing quality. In this study, we adopted the framework of coherence proposed by Halliday and Matthiessen (2004), who claimed that two zones, namely, grammatical (conjunction, reference, ellipsis, and substitution) and lexical (synonymy, hyponymy, repetition, and collocation), work together to help to contribute to a cohesive and unified whole of a text. Put differently, the use of different word forms serves to trigger certain links between elements, i.e., lexis, which in turn enable readers to make semantic inferences to capture the intended meaning in a written text. In this respect, the presence of lexical resources does not simply point to the word knowledge of learners, but more importantly, it provides semantic relations and guidance for the readers to navigate through some previously introduced or subsequent lexical items to incorporate the input sentence with all the possible contextual assumptions; thus, the text is presented to the readers as a coherent whole. As a result, the precise nature of the variety of word choices is closely associated with functions (i.e., cohesion) that are relevant to the content, without which choice of words, in isolation, would be pointless and does not make any sense. A random aggregation of diverse and sophisticated word forms in a text can, by no means, be regarded as lexical complexity. What really matters is how well these words work in concert. Thus, this study attempts to propose a revised construct of coherence (function)-based lexical complexity to predict variations in proficiency level using five lexical markings: repetition, synonymy, hypernymy, collocation, and frequency.

In the first place, a major dimension along which lexical diversity can be captured is variations of the lexical type-token ratio (TTR). TTR is fundamentally a way of measuring lexical repetition, and an acknowledged weakness is the intervening effect of text length. To compensate for sample size, a measure of textual lexical diversity (MTLD) was used in this study to deal with “the range of different words in a text” (McCarthy and Jarvis, 2010, p. 381). MTLD basically targets the extent to which words are repeated, and repetition can be considered as an indicator of cohesive devices that correlate well with writing quality. It follows that MTLD helps to enhance the understanding of a reader of what a writer intends to convey and highlights the information to which attention should be paid. Therefore, it is of great necessity to repeat certain lexical items in a given writing task, and repeated exposures to a lexical element would benefit subsequent retrieval of relevant information. It is worth pointing out that the term *repetition* is not equivalent to *redundancy*, as “repetition in its purest sense is an objective phenomenon, whereas redundancy is fundamentally subjective…,” which is “in the sense of being grounded in human perception” (Jarvis, 2013, p.20). It is also worth stressing that both inflectional and derivational variants suggest the same lexical item (recognized as repetition) (Halliday and Matthiessen, 2004), for instance, *dine* and *dinner, rational* and *rationalize*. To keep a properly narrow focus, MTLD only analyzes repetition that features occurrences of identical words; measurement of lexical variants will be discussed below in the domain of morphological complexity.

Apart from straightforward repetition, lexical complexity can be realized through the use of synonyms. Though having a differing denotational or connotational meaning, synonym plays a vital role in lexical production of learners and helps the reader to track and identify the interactions of words in a text. Patterns of synonyms can take the form of both nouns and verbs, for instance, *letter* and *missive, begin* and *start*.

Another relationship among word forms that examine the depth of lexical knowledge of learners is manifested in hypernymy. Hypernymy indicates a type of semantic relation of being a superordinate; it represents a generic term compared with a specific term (hyponym). In this sense, L2 learners with a high level of proficiency tend to use a subcategory rather than a general class. More specifically, specific terms for a broader term would indicate more fine-grained semantic properties in which a more general concept is lacking; the higher the hypernymy rating, the more complex lexical connections in a text. Such hierarchical relationships, therefore, can be used as measures of lexical sophistication that will be predictive of writing quality (Guo et al., 2013).

L2 lexical networks can also be tracked by indices of collocation (contextual distinctiveness). This sense relationship is based on a particular association between words that work together to create relationships in written contexts. For instance, the presence of *dine* would trigger the co-occurrence of the *restaurant*. Accordingly, “word co-occurrence is a strong predictor of word learning and processing (i.e., a word's contextual distinctiveness)” (Kim et al., 2018, p.122), and co-occurring words can be suggestive of contexts in which semantic representations of a word can be traced (McDonald and Shillcock, 2001).

Corpus-driven word frequency serves as our final selected measure to investigate a variety of words that are related to lexical production. Although in a broad sense frequency is not attributive to the internal organization of lexically cohesive relations, it provides a kind of checklist to examine the frequency of lexical items learners have used in a text. The reason is that whatever their lexical selections are, they all fall within the domain of the lists of available words in a language. In addition, studies have shown that there is a significantly positive correlation between word frequency and writing quality (Laufer and Nation, 1995; Morris and Cobb, 2004). Furthermore, employment of low-frequency words has suggested advancement in proficiency level (Lindqvist et al., 2011), as L2 learners are subjected to more exposure to high frequency words (Laufer, 1997). For this, the measure of word frequencies serves as an alternative to revealing the degree of informativeness reflected in word forms, and an indicator of the size of vocabulary knowledge.

Two morphological measures, encompassing both inflectional and derivational variations, were used in this study to examine how L2 learners deploy the internal structures of words across proficiency levels and topics: Types per Family (T/F) index (Horst and Collins, 2006), and morphological complexity index (MCI) (Brezina and Pallotti, 2015; Pallotti, 2015). Specifically, the T/F index targets the types-per-family ratio, aiming to capture the proportions of morphologically different word types. For instance, *golf*, *golfer, golfs, golfed*, and *golfing* belong to one word family with five word types. By analyzing the kinds of words learners have used from word frequency bands, the T/F index would indicate counts of word families that serve to distinguish between proficiency levels in terms of both derived and inflected words used in written texts. It is worth noting that word knowledge that is reflected in T/F also points to a subcategory of the aforementioned lexical measure *repetition*.

The other metric, MCI, examines the diversity of verb inflections as well as the number of varying inflectional words. It is worth noting that MCI only touches on verbal inflection without taking into account the number or ratio of the varied lexis; its exponence is, therefore, different from that of the T/F index and can be used to complement the assessment of morphological competence of a learner.

### Tools

Altogether, six automated coding instruments were used in this study: L2 Syntactic Complexity Analyzer (L2SCA; Lu, 2010), the Tool for the Automatic Analysis of Lexical Sophistication (TAALES) (Version 2.2); Kyle and Crossley, 2015); Tool For The Automatic Analysis Of Lexical Diversity (TALLED, Version1.3.1) (Kyle et al., 2021, in press), Tool For The Automatic Analysis Of Cohesion (TAACO 2.0) (Crossley et al., 2019, in press), VocabProfile^3^, and Morpho complexity tool (MC tool; Brezina and Pallotti, 2015; Pallotti, 2015). L2 Syntactic Complexity Analyzer produces eight different but interrelated syntactic measures at the sentential, clausal, and phrasal levels; TALLED measures repetition in the realm of lexical complexity by calculating MTLD that targets the correctly transformed lexical type-token ratio in a text; TALLES calculates a wide range of indices linked to lexical sophistication: word frequency, hypernymy, and contextual distinctiveness, which correspond to frequency, hypernymy, and collocation, respectively, in the lexical zone; TAACO taps into diversity in synonym that consists of both noun and verb synonyms. As for the measurement of morphological indexes, VocabProfile is a free online vocabulary analysis tool that calculates the T/F ratio (Horst and Collins, 2006): word families in terms of the diversity of both inflectional and derivational diversity. In addition, the MC tool is used to compute the number of inflectional morphological exponents to examine the inflectional diversity (verbs only), with MC index = [(within-subset variety + between-subset diversity/2) −1]. This study adopts the parameters of segment size “10” with random trials “100” (index V100, henceforth). That is, the MC tool draws subsamples of 10 forms of verbs (tokens), along the morphological dimension of complexity with 100 random trials, as the segment size in a random way to calculate inflectional exponences. A breakdown of the selected measures and tools of calculation is given in Table 3.

**Table 3:** Summary of the 15 selected measures and tools of calculation.

	**Measures**	**Code**	**Tools of calculation**
Syntactic	Mean length of clause	MLC	
Complexity	Clauses per sentences	C/S	
	Dependent clauses per T-unit	DC/T	
	Clause per T-unit	C/T	L2SCA
	T-units per sentence	T/S	
	Coordinate phrases per T-unit	CP/T	
	Complex nominals per T-unit	CN/T	
	Verb phrase per T-unit	VP/T	
Lexical	Word frequency	Frequency	TAALES
Complexity	Measure of textual lexical diversity	MTLD	TALLED
	Synonym	Synonym	TAACO
	Hypernymy	Hypernymy	TAALES
	Contextual distinctives	Collocation	TAALES
Morphological	Morphological complexity index (MCI)	V100	Morpho complexity tool
Complexity	Types per Family index	T/F	VocabProfile

## Results

### Relationships Between Complexity-Related Measures and Human-Rated Essay Quality

To explore what measures correlate with a human judgment of writing quality (i.e., between proficiency levels), Pearson correlation coefficients were calculated between the values on each measure and the human-rated score level (groups *medium* and *high*). Table 4 summarizes the correlations of individual complexity measures with human-rated scoring. As indicated in Table 4, no quantitative variables are linearly related except index V100, suggesting that only the use of V100 is significantly different across writing proficiency levels, with *r* = 0.133, *p* < 0.05 (0.028). In other words, only one morphological measure out of all the 15 complexity measures reflects a positive association between the groups *medium* and *high*, as a change in the use of V100 will have an effect on essay quality. The results of correlation coefficients are contrary to what is hypothesized: when score level is taken into account, measures along the syntactic, lexical, and morphological dimensions between the groups *medium* and *high* tend to be simultaneously greater than, or simultaneously less than, their respective means to be positive.

**Table 4:** Correlations of 15 complexity measures with human-assigned score level.

	**Measures**	***p***	**Sig. (2-tailed)**
Syntactic	MLC	0.061	0.315
Complexity	C/S	0.093	0.123
	DC/T	0.092	0.129
	C/T	0.097	0.107
	T/S	−0.059	0.329
	CP/T	0.017	0.780
	CN/T	0.043	0.473
	VP/T	0.096	0.113
Lexical	Frequency	−0.022	0.716
Complexity	MTLD	−0.043	0.473
	Synonym	0.003	0.959
	Hypernymy	−0.056	0.358
	Collocation	0.098	0.106
Morphological	V100	0.133	0.028^*^
Complexity	T/F	0.013	0.835

* *Correlation is significant at the 0.05 level (two-tailed)*.

As the next step after correlation analysis, multiple linear regression (MLR) was used to explore the strength of the relationship between a dependent variable (DV) (score level) and one or more independent variables (IVs), since the outcome variable (the value of a DV) is assumed to be predicted by each of the individual measure (IVs, the predictor variables), that is, how much of the variation regarding human scoring can be explained by complexity measures. Prior to performing MLR analysis, assumptions for regression such as linearity, normality, multicollinearity, and homoscedasticity, have been checked and indicated that these assumptions are met. It is worth noting that the use of regression is aimed to determine differences between two nominal variables (groups *medium* and *high*); therefore, dummy-coded variables (known as categorical variables) have been created. Table 5 presents an overview of the results of MLR.

**Table 5:** Coefficients^a^.

**Model**		**Unstandardized coefficients**		**Standardized coefficients**	***t***	**Sig**.
		**B**	**Std. Error**	**Beta**		
1	(Constant)	0.417	0.232		1.800	0.072
	MLC	0.003	0.003	0.033	0.912	0.362
	C/S	−0.067	0.097	−0.136	−0.688	0.492
	DC/T	0.147	0.107	0.186	1.371	0.171
	C/T	−0.179	0.150	−0.276	−1.196	0.232
	T/S	0.032	0.195	0.017	0.164	0.870
	CP/T	0.120	0.056	0.077	2.155	0.031
	CN/T	0.054	0.024	0.117	2.250	0.025
	VP/T	0.002	0.044	0.004	0.043	0.966
1	(Constant)	−0.984	0.182		−5.399	0.000
	Frequency	0.002	0.010	0.010	0.252	0.801
	MTLD	0.006	0.001	0.179	5.217	0.000
	Synonym	0.011	0.002	0.190	6.180	0.000
	Hypernymy	0.197	0.039	0.176	4.996	0.000
	Collocation	−0.003	0.010	−0.010	−0.317	0.752
1	(Constant)	−0.426	0.461		−0.925	0.355
	V100	0.071	0.013	0.180	5.467	0.000
	T/F	0.353	0.420	0.028	0.841	0.401

a *Dependent variable: group = 2*.

As for the prediction of scoring from the relative contribution of each of the eight syntactic complexity measures, dummy-coded regression analysis shows that *F*(8, 993) = 4.211, *p* < 0.001 (0). *R* is.181, suggesting a low degree of correlation, and the *R*^2^ value is 0.033, indicating that 3.3% of the total variation in the dependent variable (proficiency level, i.e., human-rated score level) can be explained by the independent variables (syntactic measures). This is a very small good fit for the data. For coefficients of each of the predictors, only CP/T and CN/T had significant positive regression weights (with *p* = 0.031 and 0.02, respectively), indicating that test-takers with higher values on the two measures were expected to receive better writing scoring from human expert raters. As for the six other syntactic measures, they did not contribute significantly to the multiple regression model, indicating no statistically significant differences in proficiency level.

Turning to lexical measures, the MLR analysis indicates that the values of *R* and *R*^2^ are 0.303 and 0.092, respectively, suggesting that lexical measures explain 9.2% of the variance in the human-rated essay quality. This is also small goodness of fit. The *p*-value for the *F* test [*F*(5, 996) = 20.141] is 0, indicating that complexity at the lexical level statistically significantly predicts human scoring. As for independent variable coefficients, three out of the five measures, namely, synonym, hypernym, and MTLD, added statistically significantly to the prediction, with *p* < 0.001 (0, 0, and 0, respectively).

The results of MLR for the morphological complexity with two predictors (i.e., V100 and T/F) produced *R* = 0.191 and *R*^2^ = 0.036, indicating a weak relationship between the predictor variables and the outcome variable. In other words, the regression model is a relatively weak predictor of the outcome (proficiency level), and 3.6% of the variance in the data can be explained by the predictor variables (two morphological indexes). As for the results of analysis of variance, *F*(2, 999) = 18.821, *p* < 0.001 (0), indicating that the model was a significant predictor of human evaluation of quality writing. A closer look at the values of coefficients reveals that only V100 has a significant positive weight on the performance of test-takers between proficiency levels.

Furthermore, an independent sample *t* test with 95% confidence interval was carried out to explore which measures yielded significant differences between the two unrelated subsets: group *medium* and group *high*. To avoid Type 1 error, a Bonferroni correction was applied, which resulted in corrected alpha values of 0.00625 (0.05/8 = 0.00625),0.001 (0.05/5 = 0.001), and 0.025 (0.05/2 = 0.025) for complexity measures at the syntactic, lexical, and morphological levels, respectively. Given the results of the *t*- test, five out of the eight syntactic measures are found to be significantly different between proficiency levels, with C/S (t(564.333) = 3.815, *p* = 0), DC/T (t(1000) = 2.435, *p* = 0.015), C/T (t(514.048) = 2.906, *p* = 0.004), T/S (t(639.363) = 2.346, *p* = 0.019), and CP/T (t(1000) = −2.372, *p* = 0.018). As for lexical complexity, test takers displayed significant differences across each group in use of synonym (t(1000) = −4.481, *p* = 0), hypernym (t(1000) = −6.104, *p* = 0), and MTLD (t(1000) = −5.642, *p* = 0), respectively, with the other two indexes (frequency and collocation) suggesting no proficiency effect on their lexis decisions. Finally, both indexes of morphological complexity were significantly different between variances of the two proficiency groups, with V100 (t(1000) = −6.078, *p* = 0) and T/F (t(1000) = −2.744, *p* = 0.006).

As a complement to the *t*-test, effect sizes were also calculated to determine the “size” of the differences between group means. Table 6 summarizes the values of Cohen's D. Considering the results extracted from the independent *t*-test, it is clear from Table 6 that although there are considerable differences between the groups *medium* and *high* with regard to the use of hypernym and V100, the observed standardized mean differences were often associated with small effect sizes, representing a relatively moderate differentiation between the two groups on a given variable. It is also suggested that despite the statistically significant differences in other measures as the aforementioned in the *t*-test, namely, C/S, DC/T, C/T, T/S, CP/T, MTLD, synonym, V100, and T/F, such differences are indicative of trivial effect sizes that are negligible because of their very small magnitude of effect when the two groups are compared.

**Table 6:** Statistics of complexity measures and effect sizes.

	**Measures**	**Group**	**Mean**	***SD***	**Cohen's D**
Syntactic	MLC	*Medium*	10.0219	5.1542	0.12
Complexity		*High*	10.5780	4.0070	
	C/S	*Medium*	2.3716	0.9383	0.26
		*High*	2.1419	0.8147	
	DC/T	*Medium*	0.9398	0.5596	0.18
		*High*	0.8428	0.5713	
	C/T	*Medium*	2.0402	0.6934	0.21
		*High*	1.9020	0.6635	
	T/S	*Medium*	1.1589	0.2443	0.18
		*High*	1.1249	0.1875	
	CP/T	*Medium*	0.3964	0.2882	0.14
		*High*	0.4448	0.2876	
	CN/T	*Medium*	2.1245	1.0007	0.02
		*High*	2.1406	0.8891	
	VP/T	*Medium*	2.8276	0.9472	0.13
		*High*	2.6927	0.9920	
Lexical	Synonym	*Medium*	14.2912	7.1209	0.3
Complexity		*High*	16.6855	8.5740	
	Frequency	*Medium*	8.3571	1.7884	0.05
		*High*	8.4443	1.4989	
	Hyponymy	*Medium*	3.8823	0.3994	0.44
		*High*	4.0518	0.3721	
	Collocation	*Medium*	9.4921	1.4786	0.01
		*High*	9.4668	1.3261	
	MTLD	*Medium*	55.2154	13.3558	0.39
		*High*	60.6953	14.6365	
Morphological	V100	*Medium*	4.0772	1.1222	0.43
Complexity		*High*	4.5519	1.0511	
	T/F	*Medium*	1.1309	0.0352	0.28
		*High*	1.1377	0.0337	

### Effects of Topic on Linguistic Complexity

To examine how within-discourse-mode topics (i.e., argumentative essay) influence the production of complexity measures by test takers at the same score level, one-way between-subjects repeated measures ANOVA tests with *post hoc* analysis using Turkey HSD were performed to unveil whether there were statistically significant differences across eight topics. Table 7 shows the results of the effects of the topic as well as the sizes of topic effect (measured with eta2).

**Table 7:** Topic effects and effect sizes (within-groups).

		**Group** ***medium***			**Group** ***high***		
	**Measures**	***F*(7,704)**	***p***	**η2**	***F*(7,267)**	***p***	**η2**
Syntactic	MLC	1.824	0.800	0.018	1.062	0.388	0.027
Complexity	C/S	1.379	0.211	0.014	0.285	0.959	0.007
	DC/T	1.505	0.162	0.015	0.888	0.516	0.023
	C/T	1.323	0.237	0.013	0.776	0.605	0.020
	T/S	0.695	0.676	0.007	1.374	0.216	0.035
	CP/T	12.426	0.000^***^	0.110	7.846	0.000^***^	0.171
	CN/T	6.075	0.000^***^	0.057	2.693	0.010^**^	0.066
	VP/T	2.661	0.010^**^	0.026	0.284	0.960	0.007
Lexical	Frequency	25.531	0.000^***^	0.202	11.458	0.000^***^	0.231
Complexity	MTLD	9.448	0.000^***^	0.086	5.329	0.000^***^	0.123
	Synonym	2.934	0.005^**^	0.028	0.681	0.688	0.018
	Hyponymy	45.085	0.000^***^	0.310	13.353	0.000^***^	0.259
	Collocation	13.586	0.000^***^	0.119	10.38	0.000^***^	0.214
Morphological	V100	5.552	0.000^***^	0.052	2.764	0.009^**^	0.068
Complexity	T/F	8.923	0.000^***^	0.081	2.653	0.011^*^	0.065

* *p < 0.05*;

** *p < 0.01*;

*** *p < 0.001*.

As can be seen, for test-takers from the group *medium*, there was a significant effect of topic on the use of complexity measures at the *p* < 0.05 level. Three out of the eight syntactic measures and all the five lexical and two morphological measures differed significantly across eight topics. No significant differences were found between topics regarding five syntactic measures, namely, MLC, C/S, DC/T, C/T, and T/S. Similar findings were reported from the group *high*, where two syntactic measures (CP/T and CN/T), four measures of lexical complexity (frequency, MTLD, hypernymy, and collocation), and two morphological measures (V100 and T/F) showed significant differences across topics. Taken together, the findings suggest that the topic does affect the ability of test-takers to select appropriate complexity measures, thus contributing to the variation and overall production of linguistic devices.

As for the magnitude of effects, values of eta squared of CP/T, CN/T, and VP/T in the syntactic layer from the group *medium* correspond to 0.11, 0.057, and 0.026, respectively, suggesting a large, medium, and small effect size, respectively. While for lexical and morphological complexity, the topic was found to have a relatively large effect on the lexical use of hypernymy, frequency, and collocation (values correspond to 0.31,0.202, and 0.119, respectively), the other two lexical measures, and two morphological ones that revealed statistical significance between proficiency levels were found to statistically differ with small to medium topic effects (η^2^ ranging from 0.028 to 0.086). As for topic effects within the group *high*, the results indicated that three lexical measures out of the overall eight complexity measures that were observed to show statistically significant differences reported large effect sizes (hypernymy: η^2^ = 0.259; collocation: η^2^ = 0.214; frequency: η^2^ = 0.231). Small to medium effects sizes were found in terms of the other five linguistic measures, namely, CP/T and CN/T for complex syntactic devices, MTLD for lexical complexity, and V100 and T/F for the complexity of morphological items, indicating that although these measures had statistical differences in proficiency, their effect sizes associated with them did not represent strong predicative strength.

Furthermore, a two-way between groups ANOVA was carried out to measure whether and to what extent two main effects, i.e., proficiency and topic, would explain the variances in one interaction effect (complexity measures). Table 8 presents the results of the two-way ANOVA.

**Table 8:** Results of two-way ANOVA.

	Topic*Group Effects			
	**Measures**	***F*(7,986)**	***p***	** $\eta_{\text{p}}^{2}$ **
Syntactic	MLC	0.488	0.844	0.003
Complexity	C/S	0.359	0.926	0.003
	DC/T	0.284	0.960	0.002
	C/T	0.228	0.979	0.002
	T/S	1.023	0.413	0.007
	CP/T	0.597	0.759	0.004
	CN/T	0.229	0.979	0.002
	VP/T	0.462	0.862	0.003
Lexical	Frequency	2.190	0.261	0.009
Complexity	MTLD	1.272	0.846	0.003
	Synonym	0.486	0.168	0.010
	Hyponymy	1.487	0.291	0.009
	Collocation	1.215	0.033	0.015
Morphological	V100	1.677	0.111^*^	0.012
Complexity	T/F	0.750	0.629	0.005

* *p < 0.05*.

As for the statistically significant effects of topic and proficiency on the output of linguistically complex measures by test takers, the results indicated that the deployment of almost all of the complexity measures was found not to be influenced by the two independent variables (proficiency and topic), with the only exception that frequency showed a significant interaction effect between proficiency^*^topic and the lexical production of test-takers, with a small effect size of $\eta_{\text{p}}^{2}$ equaling to a value of 0.015 [*F*(7, 986) = 2.19, *p* = 0.033].

## Discussion

This study aims to provide an extended account for validating complexity measures that feature prominently in studies of L2 writing. Specifically, we tapped into changes in language production in terms of the three distinct constructs of linguistic complexity among Chinese EFL learners across different topics and proficiency levels using writing samples selected from TOEFL Corpus 11, a new large-scale corpus of non-native English writing in a high-stakes English proficiency test.

As for research question 1, “relationships between linguistic complexity and human-rated score level”, the overall findings, which target the predicative power and value of the selected complexity measures, suggested weak validity evidence. First, only one morphological measure, V100, was found to show a linear correlation between the groups *medium* and *high*. This is in line with prior findings that indicated that MCI, which is geared toward measuring the variability in verbal inflection, can be indicative of the proficiency of learners (De Clercq and Housen, 2016; Brezina and Pallotti, 2019). However, the 14 other measures along the syntactic and lexical dimensions of complexity failed to discriminate between test-takers at varying proficiency levels. In this regard, the results cast doubt on previous studies concerning the positive role these two complexity constructs play in essay quality. For instance, “reliance on phrasal structures, especially complex phrases with phrasal modifiers” generally characterizes better academic compositions (Biber et al., 2011, p. 192). Complexity at the level of subordination and coordination correlated significantly with high quality of writing performance (Flahive and Snow, 1980; Homburg, 1984; Grant and Ginther, 2000). As for lexical complexity, our finding lent further support to the claim that lexical knowledge failed to contribute to the improvement in overall writing proficiency (Malvern et al., 2004; Yu, 2010; Bulté and Housen, 2014; Pietila, 2015).

Second, although the MLR analysis revealed statistically significant differences in a number of measures, their predicative strength of the explanatory variables had proven to be less strong than expected. That is, a small proportion of the variation in human rating can be explained by the variation in the selection of linguistic features by the test takers.

Third, with regard to the results of the independent *t*-test, our study confirmed the contribution of several linguistic measures to the holistic human scoring. This is evidenced by the five syntactic measures (C/S, DC/T, C/T, T/S, and CP/T), three lexical measures (synonym, hypernymy, and MTLD), and two indexes of morphological complexity (V100 and T/F), all of which appeared to serve as good indicators of EFL writing proficiency. However, most of the measures with significant differences only have small to medium size effects when the two group means (*medium* and *high*) were compared, indicating relatively moderate relationships between the structural dimensions of linguistic devices of test takers and human judgment of essay quality. In all, proficiency among Chinese EFL learners may not signal a discriminative power in their language production, and complexity in the syntactic, lexical, and morphological dimensions does not account for a major contribution to human score levels.

For research question 2 “effects of topic on the use of complexity measures,” the results of within-proficiency topic effects from the group *medium* indicated that significant topic effects were observed for the majority of the complexity measures, including three out of the eight syntactic measures (i.e., CP/T, CN/T, and VP/T) and all the lexical and morphological constructs of complexity. In other words, within the same genre of argumentative writing, the values of most of the diversified forms of complexity were found to be significantly different in the group *medium* across eight prompts. Topic does play a role in the use of linguistic devices by test takers. Similar topic relevance was also found in the group *high*, with a total of eight measures displaying significant differences across topics (excluding six indexes of syntactic complexity, i.e., MLC, C/S, DC/T, C/T, T/S, and VP/T and one lexical measure, i.e., synonym). The results, on the one hand, further supported the findings with respect to the topic effects on the textual features of language production (Reid, 1990; Spaan, 1993; Robinson, 2007, 2011). For instance, strong topic effects have been found on average word length and word frequency out of a very limited number of lexical indices in the study of Yoon (2017). Topic familiarity also affects the choice of lexical measures of learners (Skehan, 1998; Yang and Kim, 2020), as different topics require different reasoning demands, thus generating different complexity measures. On the other hand, it also provides inconsistent evidence regarding the effects of the topic on syntactic variances (e.g., Hinkel, 2002; Yang et al., 2015). For instance, significant differences were observed in syntactic complexity at the local levels like coordination, subordination, and noun-phrase complexity, as well as length-related variables (Yang et al., 2015). Generally, Chinese EFL learners tend to be influenced by their level of proficiency and varied topics when lexical and morphological features are taken into consideration, in addition to a limited impact of the topic on a few syntactic measures (only CP/T and VP/T). In other words, dimensions of the topic do not motivate much change in the diversity and variations of the syntactic performance of Chinese learners. Syntactic variety cannot be used as a benchmark for illustrating the differences in writing performance among EFL learners at a higher level of proficiency.

In the analysis of between-proficiency topic effects on complexity, we have found that when a combination of both proficiency and topic effect was taken into account, no significant interaction effect was observed on linguistic performance of learners, with the only exception that the index of frequency yielded a statistically significant interaction. The group means of all the selected complexity features did not represent significant differences in association with the interaction of two variables: proficiency level and topic.

## Conclusion

This study aimed to examine what measures would predict highly trained human rating in high-stakes tests along the three dimensions of linguistic complexity among Chinese EFL learners. It also touched upon the prompt (topic)-related variations that would impact the presence or absence of linguistic features in essays written by learners. Our analysis revealed conflicting findings with regard to the relationships between complexity measures and writing performance judged by human raters. One manifestation lies in the only morphological index of V100 that demonstrates a positive significant correlation between the groups *medium* and *high*. For this, this study casts doubt on the construct validity of complexity measures, as the correspondence between the use of linguistic complexity and human scoring is considered negligible. In the meantime, the results of the independent *t*-test indicated that there was a statistically significant difference regarding two-thirds of the total 15 measures when the means of individual measures between two groups were compared. In general, our findings suggested that complexity plays a moderate but not essential role in writing assessment.

In addition, this study also yielded conflicting findings of topic effects. On the one hand, mediating effects from task variables (referred to as topics in this study) have been observed within the same proficiency level. On the other hand, no favorable findings have been achieved with respect to the interaction effects of a combination of both proficiency and topic on the use of structural complexity in learners' language production (the only exception lies in one lexical index: frequency). This makes sense, however. Although the eight prompts fall within the same discourse mode of argumentative writing, they do differ in terms of levels of familiarity, cognitive and reasoning demand, experiences, and so on. Another factor is that the analysis is based on the between-subjects design as the TOEFL11 Corpus does not comprise essays that are written by the same test takers; a within-subjects one that is designed to balance out participant-level errors would otherwise affect the results of the current observations.

This study also has implications for both EFL instructors and learners, as well as human raters. First, due attention should be paid in instructional settings concerning the association between writing quality and language production. Second, explicit and targeted teaching is of vital importance in classrooms in that the accurate output of linguistic features is contingent upon an in-depth understanding of the learnability issues in linguistic performances of students, as well as well-designed curriculum and course materials. Third, it is suggested that learners should raise their awareness of how to incorporate correct and appropriate forms of linguistic features into their academic writings, as the acquisition and development of complexity measures must take accuracy into consideration. Finally, as for human raters, the evaluation of writing quality that is heavily based on linguistic features in academic settings cannot fully represent the writing proficiency of learners. Raters should not be positively affected by the presence of complexity, since they must take into account the correct use of measures extracted from linguistic complexity. In addition, when evaluating writing responses and assigning a score, raters should adopt a holistic rating scale in a consistent manner by integrating complexity with other components of writing.

It is worth noting that this study only tapped into complexity measures reflected in the corpus data of learners without taking on issues, such as grammar, accuracy, and misspelling, that may influence the results in writing assessment. It is believed that any correction or editing of these errors prior to statistical analysis would impact the perception of essay quality by human judges. It is also important to note that this study only investigated the role individual complexity dimension had played in accounting for a large proportion of scoring variances in a high-stakes test, there would be a more precise and fine-grained understanding when the three dimensions of linguistic complexity are modeled together or with another (for instance, examination of the relationships between any two of the three dimensions and human-rated writing quality). In this regard, the results in this study may not be generalized and would invite future studies for further elaboration and new findings. As for future research in this direction, how the aforementioned two dimensions of complexity, namely, absolute and relative complexity (Bulte and Housen, 2012), interact to influence writing quality would be the follow-up field of investigation in second language studies. In addition, researchers should dig into details on specific categories within each dimension of linguistic complexity to capture a better and systematic understanding of the employment of linguistic features among learners of English.

## Data Availability Statement

The original contributions presented in the study are included in the article/supplementary material, further inquiries can be directed to the corresponding author/s.

## Author Contributions

LQ designed the study and wrote the whole manuscript. YC edited the manuscript. YZ proofread the manuscript. All authors contributed to the article and approved the submitted version.

## Conflict of Interest

The authors declare that the research was conducted in the absence of any commercial or financial relationships that could be construed as a potential conflict of interest.

## Publisher's Note

All claims expressed in this article are solely those of the authors and do not necessarily represent those of their affiliated organizations, or those of the publisher, the editors and the reviewers. Any product that may be evaluated in this article, or claim that may be made by its manufacturer, is not guaranteed or endorsed by the publisher.

## Footnotes

^1^Based on the *Test and Score Data Summary for TOEFL iBT® Test* (2010-2019) (https://www.ets.org/toefl).

^2^ https://www.ets.org/s/toefl/pdf/toefl_writing_rubrics.pdf

^3^Freely available at http://www.lextutor.ca/vp/comp (accessed in April, 2021).
